# Supplementary material for: The effects of physical exercise, parent-child interaction and peer relationship on adolescent depression: An empirical analysis based on CEPS data
Source: PLoS One. 2024 Dec 4;19(12):e0313489. doi: 10.1371/journal.pone.0313489 (PMC11616870; doi:10.1371/journal.pone.0313489)
Supplement: Attachment — Submitted filename: Response to Reviewers.docx [file pone.0313489.s001.docx]

Dear Editor and Reviewers,

I would like to express my sincere gratitude for the opportunity to revise and resubmit our manuscript, " The effects of physical exercise, parent-child interaction and peer relationship on adolescent depression: an empirical analysis based on CEPS data", for consideration for publication in PLOS ONE. The insightful comments and suggestions from the reviewers have greatly contributed to enhancing the quality and clarity of our research. We have carefully addressed each point raised during the review process and made substantial revisions as detailed below.

**Reviewer #1:** The paper is well-written and presents a highly relevant research question. My main concern regards the causal interpretation of the estimation results. As a consequence of their personality, different children may have different life attitudes, and this is correlated with practicing exercise, being in a good mood and having good relationships with parents and peers.

RESPONSE: Thank you for your careful reading and positive comments on this article! Below, we respond to your comments line by line, and we welcome any further suggestions you may have.

1. While the paper tries to address the endogeneity concerns with a propensity score analysis, this approach is not valid to deal with endogeneity as it cannot control for unobserved variables (i.e. children’s personality).

RESPONSE**:** We have carefully considered your suggestions and taken steps to address the endogeneity concerns raised. Specifically, we conducted the Oster (2019) robustness test, as you recommended, to assess the sensitivity of our estimation results to the presence of unobserved components. This test has been added to the manuscript on line 255. The results of this analysis provide further support for our original findings, indicating that our estimates are robust to the potential influence of unobserved variables.

We believe that this additional analysis significantly strengthens our paper by providing further evidence for the causality of the relationships we investigated. We are grateful for your guidance in this matter and for suggesting such a valuable test to enhance our research.

Regarding the second approach you suggested, employing instrumental variables, we acknowledge that this could be a powerful tool to address endogeneity concerns. However, identifying valid instruments in our context is challenging and requires further research and data collection. We will continue to explore this option and consider it for future work.

Once again, thank you for your insightful comments and suggestions. We have incorporated your feedback into the manuscript and believe that it has greatly improved the quality and robustness of our research. We look forward to the next steps in the publication process.

**Reviewer #2:** The manuscript explores an important topic: adolescent depression and the roles physical exercise, parent-child interaction, and peer relationships play in mitigating its effects. Using data from the China Education Panel Survey (CEPS), the authors present a well-structured empirical analysis that examines the associations and mediating roles between these variables. The study contributes valuable insights into the prevention of adolescent depression, especially in the context of physical activity. However, there are several areas where the manuscript could be improved, particularly in terms of methodological transparency, discussion of limitations, and the clarity of the presentation.

RESPONSE: Thank you very much for your thorough review and constructive comments. We appreciate your efforts to improve the quality of our manuscript and have carefully addressed each of your concerns in the revised version.

1. While the manuscript outlines the key variables and control variables used, it lacks detailed justification for some variable choices, such as the thresholds for categorizing physical exercise participation. Providing more clarity on these criteria would improve the transparency of the methodology.

RENSPONSE: We deeply apologize for the lack of detailed reasons for selecting some variables in the manuscript you pointed out, especially the threshold setting for the classification of physical exercise participation. To enhance the transparency of the method, we hereby clarify and provide a detailed explanation of our method for calculating the average daily exercise time of adolescents.

The data on physical exercise participation was sourced from the China Education Panel Survey (CEPS), which asked participants about the number of days they engaged in physical exercise per week and the average duration of each exercise session. We calculated the average daily exercise time using the formula: average daily exercise time = (weekly exercise days × average daily exercise duration / 7).

Regrettably, due to the limitations of the existing database, we were unable to classify exercise intensity based on specific thresholds. We acknowledge this as a limitation of our study and will include a discussion of the issue in the limitations section of the manuscript.

2. The mediation analysis is a strength of the study, but the description of the analytical approach is somewhat unclear. Expanding on the steps taken in the mediation models and explaining why certain mediators were chosen (e.g., why peer relationships were included but other potential mediators, such as academic stress, were not) would enhance the reader’s understanding.

RESPONE: Thank you for your valuable feedback. We fully understand your concerns about the description of intermediary analysis methods and agree that a more detailed explanation will help enhance readers' understanding. In this study, we chose peer relationships and parent-child interactions as mediating factors based on theoretical and empirical evidence (see references 18-21), as well as the availability of data. However, we acknowledge that the failure to include other potential mediating factors, such as academic pressure, may limit the comprehensiveness of our research. We discussed this limitation in detail in the discussion section and suggested that future research could consider including these factors to provide a more comprehensive perspective.

3. While the statistical results are presented clearly, there is limited interpretation of the magnitude of the effects. For example, the paper mentions that physical exercise has a significant effect on depression, but the practical significance of this finding is not fully discussed. Including more interpretation on the real-world impact of the findings (e.g., how much of a decrease in depression can be expected with increased physical activity) would be beneficial.

RESPONSE: We understand your concern about the explanation of the degree of impact and agree that providing more practical discussions will enhance the depth and breadth of the research. We discussed the practical significance of the impact of physical exercise on depression from line 338 to line 354. We analyzed the relationship between physical exercise and depression, and explored the potential applications of this finding in public health and clinical interventions.

We fully agree with your opinion on how much reduction in depression can be expected with increasing physical activity, which is a very good description of the results. Therefore, we have provided additional explanations in lines 185-187.

4. To strengthen the presentation of the results, it would be helpful to include effect sizes and confidence intervals for all key findings. This would give readers a better sense of the precision and reliability of the study’s conclusions.

RESPONSE: Thank you for your valuable suggestion. We fully agree on the importance of including effect size and confidence interval in enhancing the presentation of results. These statistical indicators not only provide a measure of accuracy for the results, but also enhance the accuracy and reliability of the research conclusions.

Based on your feedback, we have added a 95% confidence interval for the effect values in lines 185 to 196. This supplementary information helps readers understand the accuracy and credibility of statistical results. By providing confidence intervals, we can demonstrate the stability of research results and the credibility range of predicted values.

5. The manuscript would benefit from a more thorough discussion of its limitations. For instance, the reliance on self-reported data for depression and physical exercise could introduce bias. Moreover, the study does not address potential confounders, such as socioeconomic status or academic pressure, which might also influence adolescent depression. Addressing these issues would provide a more balanced perspective on the findings.

RESPONSE: We fully agree with your suggestion. Firstly, we are aware of the potential bias issues that may arise from self-reported data. To reduce the impact of selective bias, we used propensity score matching (PSM) method in our analysis. This method helps balance the observable features between the experimental group and the control group, thereby reducing bias introduced by data collection methods.

In addition, we also adopted the suggestion of another reviewer and included Oster test for robustness testing. This test helps evaluate the stability of our estimation results under different models, thereby enhancing the reliability of our conclusions.

Regarding the issue of potential confounding factors, we acknowledge that due to database limitations, we were unable to include all factors that may affect adolescent depression, such as socioeconomic status or academic pressure. We recognize that these factors may have an impact on the research results. We have already explained this limitation in the discussion section and suggest that future research consider these variables to provide a more comprehensive analysis.

6. While the discussion touches on some relevant studies, it would be helpful to more explicitly compare the findings of this study with previous research, particularly in terms of the magnitude and direction of effects. This would allow readers to better understand how this study advances the existing literature.

RESPONSE: We sincerely appreciate your suggestion, which provides us with an opportunity to further clarify the contribution of this study to existing literature. Based on your valuable feedback, we have made in-depth revisions to the discussion section.

In the revised version, we conducted a more detailed comparative analysis of the findings of this study with previous research. We paid special attention to the similarities and differences in the degree and direction of influence, and clearly pointed out how this study can fill the gaps in existing research or provide new perspectives for existing theories.

7. The figures and tables are helpful in summarizing the results, but some could be improved. For example, the captions could provide more detailed explanations of what the figures show and how they relate to the study’s hypotheses. Additionally, ensuring that all axes in figures are clearly labeled and that the tables present sufficient detail (e.g., confidence intervals) would improve readability.

RESPONSE: Regarding the captions, we have revised them to provide more detailed explanations of what each figure shows and how it relates to the study’s hypotheses. These enhancements should help readers better understand the results and their implications for our research.

Additionally, we have ensured that all axes in the figures are clearly labeled. This includes adding units where appropriate and making sure that all labels are easy to read and understand. We believe these improvements will greatly enhance the readability of the figures.

Sincerely,

Li Lang

College of Physical Education, Jilin Normal University
